# Next-generation sequencing of cervical DNA detects human papillomavirus types not detected by commercial kits

**DOI:** 10.1186/1743-422X-9-164

**Published:** 2012-08-16

**Authors:** Tracy L Meiring, Anna T Salimo, Beatrix Coetzee, Hans J Maree, Jennifer Moodley, Inga I Hitzeroth, Michael-John Freeborough, Ed P Rybicki, Anna-Lise Williamson

**Affiliations:** 1 Institute of Infectious Diseases and Molecular Medicine, University of Cape Town, Observatory, 7925, Cape Town, South Africa; 2 National Health Laboratory Service, Groote Schuur Hospital, Cape Town, 7925, South Africa; 3 Department of Molecular and Cell Biology, University of Cape Town, Rondebosch, 7700, South Africa; 4 School of Public Health and Family Medicine, University of Cape Town, Cape Town, Observatory, 7925, South Africa; 5 Department of Genetics, University of Stellenbosch, Stellenbosch, 7600, South Africa

**Keywords:** Human papillomavirus, Human immunodeficiency virus, Next generation sequencing, Rolling circle amplification

## Abstract

**Background:**

Human papillomavirus (HPV) is the aetiological agent for cervical cancer and genital warts. Concurrent HPV and HIV infection in the South African population is high. HIV positive (+) women are often infected with multiple, rare and undetermined HPV types. Data on HPV incidence and genotype distribution are based on commercial HPV detection kits, but these kits may not detect all HPV types in HIV + women. The objectives of this study were to (i) identify the HPV types not detected by commercial genotyping kits present in a cervical specimen from an HIV positive South African woman using next generation sequencing, and (ii) determine if these types were prevalent in a cohort of HIV-infected South African women.

**Methods:**

Total DNA was isolated from 109 cervical specimens from South African HIV + women. A specimen within this cohort representing a complex multiple HPV infection, with 12 HPV genotypes detected by the Roche Linear Array HPV genotyping (LA) kit, was selected for next generation sequencing analysis. All HPV types present in this cervical specimen were identified by Illumina sequencing of the extracted DNA following rolling circle amplification. The prevalence of the HPV types identified by sequencing, but not included in the Roche LA, was then determined in the 109 HIV positive South African women by type-specific PCR.

**Results:**

Illumina sequencing identified a total of 16 HPV genotypes in the selected specimen, with four genotypes (HPV-30, 74, 86 and 90) not included in the commercial kit. The prevalence’s of HPV-30, 74, 86 and 90 in 109 HIV positive South African women were found to be 14.6%, 12.8%, 4.6% and 8.3% respectively.

**Conclusions:**

Our results indicate that there are HPV types, with substantial prevalence, in HIV positive women not being detected in molecular epidemiology studies using commercial kits. The significance of these types in relation to cervical disease remains to be investigated.

## Background

Worldwide, cervical cancer is the third most common cancer in women, with 86% of cases and 88% of deaths occurring in developing countries [1]. In South Africa the incidence of HPV infection is high [2] resulting in cervical cancer being the leading cause of cancer-related death in women [1].

It has been conclusively established that infection with specific high-risk human papillomaviruses (HPV) is causally linked to the development of cervical cancer [3]. Other types of HPV are also aetiologically associated with anogenital warts [4]. Papillomaviruses (family *Papillomaviridae*) are small DNA viruses with double-stranded circular genomes that infect the cutaneous and mucosal epithelia. More than 200 different HPV types have been identified, with viruses in a type sharing greater than 90% sequence identity in the *L1* major capsid gene [5]. To date, full genome sequences are available for 118 HPV types. At least 40 HPV types infect the anogenital mucosa, with 12 of these classified by the International Agency for Research on Cancer (IARC) as carcinogenic to humans (Group 1), one as probably carcinogenic (Group 2A) and 12 as possibly carcinogenic (Group 2B) [6]. HPV-16 and HPV-18 are generally recognized as the most important oncogenic viruses, present in about 71% of cervical cancer cases worldwide [7]. However, several studies (for example [2,8,9]) have found significant variation in the regional contribution of HPV types to cervical cancer. Additionally, the largest worldwide HPV genotype distribution study carried out to date showed that the highest proportion of multiple HPV infections and infection with undetermined HPV types and species occurs in Africa [7]. Data on HPVs that are regionally prevalent are crucial in determining the risks associated with particular HPV types, and in informing vaccine strategies in the region. There is limited information of this kind available for South Africa.

South Africa is also faced with one of the worst Human immunodeficiency virus (HIV) epidemics in the world, with an estimated 5.63 million infected people and a higher HIV prevalence in women than men [10]. Consequently, the frequency of concurrent HPV and HIV infection is high [11-13]. HIV positive women are at an increased risk for the development of cervical disease [11]. These women have higher HPV viral loads, higher viral persistence and infection with rare and undetermined types [13,14]. Further epidemiological data for these types is required to estimate their potential oncogenic risk. HIV positive women also have a high incidence of multiple infections [14], with an observed frequency of between 50 to 80% [13,15,16]. In HIV negative populations the observed frequency is less, although higher than previously thought, at between 24.8 to 62.6% [17]. The elevated incidence of multiple HPV infections in HIV + women raises concerns over possible recombination between different genotypes and the emergence of novel pathogenic types. For example, Jiang et al., [18] recently demonstrated intratypic recombination between HPV-16 variants in a natural coinfection involving eight HPV-16 types.

Molecular diagnostic tools for HPV DNA detection in clinical samples must be able to accurately detect and genotype the specific HPV types circulating in a particular population. The majority of HPV DNA detection kits are PCR-based, targeted to known HPV types and generally to those prevalent in the developed world. These tests are therefore not suitable for the detection of rare or novel HPV types. PCR-based assays using consensus primers are, additionally, often not able to reliably detect all the HPV types involved in multiple infections [19-21], as commonly seen in HIV infected individuals.

The recent emergence of next-generation sequencing (NGS) technologies has opened up the opportunity to directly examine viral diversity in clinical specimens, without prior sequence information (reviewed in [22]. In this study we investigated the use of Illumina sequencing (sequencing by synthesis technology, http://www.illumina.com/systems/genome_analyzer_iix/technology.ilmn) to detect and genotype the HPV types present in a complex multiple infection in a cervical specimen from an HIV-infected South African woman. The HPV types detected using Illumina sequencing were compared to those detected by the Roche Linear Array HPV genotyping (LA) test on the same specimen. The prevalence of the HPV types present, but not included in the commercial kit, was also determined by type-specific PCR in the cohort of 109 HIV-infected South African women described by Moodley and co-workers [13].

## Results

Roche LA testing of specimen number HH015 from our study population identified 12 HPV types. As this specimen contained an extremely complex population of closely and distantly related HPV types it was selected for next-generation sequencing. Total DNA was extracted from specimen HH015 and, prior to sequencing, the HPV circular DNA present was enriched using a randomly primed rolling circle amplification (RCA) method.

### Illumina sequencing and analysis pipeline

**Figure 1:**
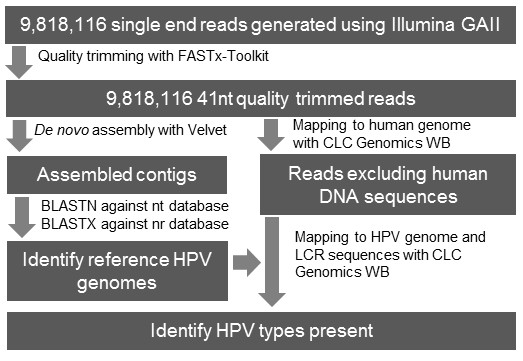
Schematic representation of the pipeline designed for the analysis of the Illumina reads and detection of HPV genotypes present.

The enriched DNA sample was sequenced using the Illumina GAII system as outlined in the Methods. A total of 9818116 reads of 76 nt in length were obtained. These short sequence reads (SSRs) were trimmed to 41 nt resulting in reads with an average quality score of 31. An analysis pipeline was designed for the detection of HPV types and performed using the SSRs (Figure 1). Briefly, to analyze the ability to assemble full HPV genome sequences from the SSR data, the reads were *de novo* assembled. The assembled contiguous sequences were then classified based on the top BLAST hits to NCBI databases. HPV sequences identified within the databases were then downloaded and used as reference sequences for mapping of the SSRs. This was done to estimate the percentage of genomes sequenced and coverage of all the HPV types present.

### Full HPV genome assembly

The trimmed SSRs were *de novo* assembled using Velvet [23] and CLC Genomics Workbench. More complete or near complete HPV genomes were assembled with Velvet than with CLC Genomics Workbench and Velvet was therefore used for all subsequent assemblies (results not shown). *De novo* assembly resulted in four contigs that represent full length HPV genomes, with top BLAST hits to HPV types 39, 40, 16, and 56 (Table 1). The coverage (or average number of times each base was sequenced) of these contigs ranged from 7466.9 for HPV-39 to 93.9 for HPV-56. A further two contigs had top BLAST hits to HPV-30 (Table 1), and together represent the complete genome (> 90%), excluding the region from bases 5302 to 5990 within the HPV-30 L2 gene. The remaining HPV types present, but not fully assembled, were identified by using all the assembled contigs greater than 100 bp in BLASTN and BLASTX searches of the NCBI databases. The HPV genome sequences identified in the BLAST searches were downloaded from the NCBI database and used as reference sequences for read mapping.

**Table 1:** BLASTN results for Velvet *de novo *assembled contigs*

**HPV type**	**Number of contigs**	**Coverage**	**Region**	**Genbank accession top hit (Identity; E-value)**
39	1	7466.9	Full length	M62849 (99;0.0)
40	1	1391.8	Full length	X74478 (99; 0.0)
16	1	1011.2	Full length	FJ610149 (99;0.0)
56	1	93.9	Full length	EF177179 (99;0.0)
30	2	43.3	5990-3276	X74474 (98; 0.0)
		49.2	3249-5302	X74474 (98; 0.0)

*Only contigs representing full or near full length genomes with a top BLASTN hit to HPV sequences are presented.

### Read mapping analysis of HPV genotypes in HH015

**Figure 2:**
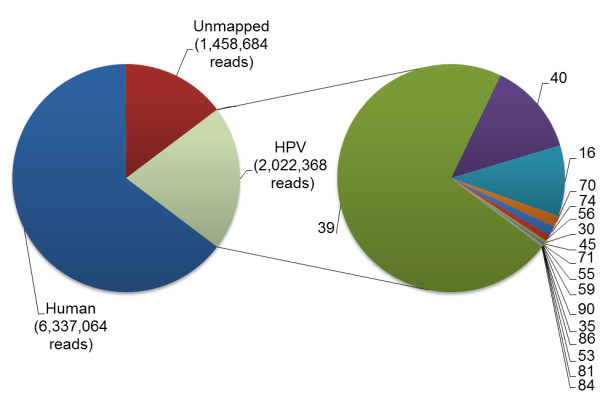
**Pie chart summary of mapping of Illumina reads from specimen HH015 to human and HPV reference sequences using CLC Genomics Workbench 4.6.1 (Global alignment, mismatch cost 2, limit 5).** Numbers on the pie chart on the right indicate HPV genotypes. The Genbank accession numbers for the sequences used as references in the mappings are given in Table 2.

Initially, any reads representing host DNA were excluded by mapping the SSRs to a human genome reference sequence. A total of 6 337 064 reads (64.5% of the total reads, Figure 2) were mapped to the human genome and excluded from subsequent analyses.

Reads excluding human DNA sequences were then mapped to the HPV reference sequences. A total of 2 022 368 reads (20.6% of total reads) were mapped to HPV genome sequences and 1 458 684 reads (14.9%) could not be mapped to human or HPV DNA sequences (Figure 2). Individual mapping to the HPV reference genomes identified HPV types 39, 40, 16, 70, 74, 56, 30, 45, 59, 71, 35, 55, 90, 53, 86, 81, and 84, in order of highest to lowest sequencing coverage (Table 2, individual mapping). HPV type 39 had the highest coverage (7604.5) and clearly represented the dominant type in the specimen. HPV types 40, 16, 70, 74, 56 and 30 had high coverage, ranging from 1384.5 for HPV-40 to 45.9 for HPV-30. Coverage greater than 10 was obtained for HPV types 45, 71 and 55, while the coverage for HPV types 59, 90, 35, 86, 53 and 81 ranged from 7.6 to 2.2. The percentage of the genome sequenced ranged from 100 to 88.6% for 12 of the HPV types identified (39, 40, 16, 70, 74, 56, 30, 45, 71, 59, 90 and 35) and from 77.9 to 51.8% for HPV types 55, 86, 53 and 81. The coverage for HPV-84 was low (1.9) and only 7.9% of the reference HPV-84 genome was sequenced.

**Table 2:** Mapping of Illumina reads generated for specimen HH015 to HPV reference genome sequences

**HPV type**	**Genbank accession of reference sequence**	**Individual mapping**			**Simultaneous mapping**		
		**Read count**^**€**^	**Coverage**^**#**^	**Percentage of genome sequenced**	**Read count**^**€**^	**Coverage**^**#**^	**Percentage of genome sequenced**
39^*^	M62849	1452838	7604.5	100.0	1450445	7592.9	100.0
40^*^	X74478	267072	1384.5	100.0	267072	1384.5	100.0
16^*^	FJ610149	204214	1059.0	100.0	204212	1059.0	100.0
70^*^	HPU21941	32187	171.0	97.6	3295	17.1	97.6
74	AF436130	27099	142.7	98.7	26802	139.3	98.7
56	EF177179	18510	97.4	100.0	18409	96.9	100.0
30	X74474	8781	45.9	99.9	8612	45.0	99.9
45^*^	EF202160	2873	15.9	94.7	774	4.0	94.4
71^*^	NC002644	2106	10.8	99.6	2088	10.7	99.6
55^*^	HPU31791	1400	14.2	51.8	587	3.1	49.2
59^*^	X77858	1342	7.6	91.2	692	3.6	91.2
90	AY057438	1136	5.9	98.0	1116	5.7	98.0
35	PPH35CG	1109	6.5	88.6	720	3.8	88.4
86	AF349909	683	5.3	66.0	680	5.3	66.0
53^*^	NC001593	682	4.6	77.9	462	2.4	77.6
81^*^	AJ620209	308	2.2	72.5	308	2.2	72.5
84^*^	AF293960	28	1.9	7.6	11	0.1	4.7

^*^ HPV types detected by the Roche Linear Array HPV Genotyping kit.

^€^ Number of reads mapped to the reference.

^#^Average number of times each base was sequenced.

The reference mapping to the HPV genomes was additionally performed using all the reference genome sequences in a single mapping (Table 2, simultaneous mapping), with random assignment of reads mapping to more than one genome. The coverage for types 39, 40, 16, 74, 56, 30, 71, 90, 86 and 81 remained equal or close to that observed when reads were mapped to the respective genomes individually. The coverage for HPV-70, 45, 55, 59, 53 and 35, however decreased significantly (underlined in Table 2) when mapped simultaneously. The coverage of HPV-70, for example decreased tenfold from 171 to 17. Although the mapping was performed at a high stringency, the difference in coverage is probably due to regions within these types having high sequence identity to regions within other HPV types present in the sample, such as parts of the region coding for the conserved L1 protein. Based on alignment of the E1-E2 and L1 genes, HPV types 70, 45 and 59 cluster with HPV-39 in the alpha-7 PV genus, HPV-55 clusters with HPV-74 in the alpha-10 genus, HPV-35 with HPV-16 in alpha-9 and HPV-53 with HPV-30 in alpha-6 [24]. As regions of high identity may lead to the possibility of over- or under-representation of an HPV type, we tested the mapping of the SSRs to a sub-genomic region that is highly variable between the HPV genomes, the long control region (LCR; Table 3).

**Table 3:** Mapping of Illumina reads generated for specimen HH015 to HPV long control region (LCR) sequences

**HPV type**	**Genbank accession of reference sequence**	**Individual mapping**				**Simultaneous mapping**			
		**Read count**^**€**^	**Coverage**^**#**^	**Percentage of LCR sequenced**	**Identity°**	**Read count**^**€**^	**Coverage**^**#**^	**Percentage of LCR sequenced**	**Identity°**
39^*^	M62849	131419	6916.8	100	100	131417	6916.7	100	100
16^*^	FJ610149	20258	998.3	100	99.8	20258	998.3	100	99.8
40^*^	X74478	19984	1147.5	100	99.6	19984	1147.5	100	99.6
56	EF177179	1940	100.2	100	99.6	1940	100.2	100	99.6
74	AF436130	1565	91.9	88.6	97.9	1560	91.6	88.6	97.9
30	X74474	789	40.9	99.2	99.2	789	40.9	99.2	99.2
71^*^	NC002644	176	10.0	92.9	100	176	10.0	92.9	100
70^*^	HPU21941	97	4.7	93.6	99.9	95	4.6	93.6	99.9
35	PPH35CG	79	4.4	84.8	99.6	79	4.4	84.8	99.6
45^*^	EF202160	68	3.7	92.5	98.9	68	3.7	92.5	98.9
59^*^	X77858	57	3.4	83.6	100	57	3.4	83.6	100
90	AY057438	55	3.6	87.4	98.6	55	3.6	87.4	98.6
55^*^	HPU31791	54	5.7	53.3	98.7	18	2.0	50.0	98.7
86	AF349909	50	4.6	55.0	98.0	50	4.6	55.0	98.0
81^*^	AJ620209	29	2.4	61.8	100	29	2.4	61.8	100
53^*^	NC001593	24	2.7	45.7	98.6	24	2.7	45.7	98.6

^*^ HPV types detected by the Roche Linear Array HPV Genotyping kit.

^€^ Number of reads mapped to the reference sequence using CLC Genomics Workbench 4.6.1 (limit 3, cost 2, global alignment).

^#^ Average number of times each base was sequenced.

° Percentage identity of the assembled contig to the reference sequence.

The SSRs were mapped to the LCR of the HPV types individually and in a single simultaneous assembly (Table 3). A high consistency was found between the two mappings for all HPV types (Table 3), with equal or near equal read counts, coverage and percentage of the LCR sequenced for all HPV types in both mappings. The coverage obtained in these mappings is then probably a more accurate representation of the relative prevalence of each HPV type in the sequenced sample. The coverage of HPV types 39, 16, 40, 56, 74 and 30 was high and ranged from 6918.8 to 40.9. HPV-71 had a lower coverage of 10 and coverage of types 70, 35, 45, 59, 90, 55, 86, 81 and 53 was the lowest from 4.7 to 2.7.No reads were found to map to the HPV-84 LCR. The percentage of the LCR sequenced ranged from 100 to 83.6% for HPV types 39, 16, 40, 56, 74, 30, 71, 70, 35, 45, 59, 90 and was lower (61.8 to 45.7%) for HPV types 55, 86, 81 and 53. This is comparable to that observed for the reference mapping to the complete genomes.

Read mappings were performed with CLC Genomics Workbench and MAQ, but the results found not to differ significantly (results not shown). The reference mappings reported here were performed with CLC Genomics Workbench (Tables 2 and 3).

### Prevalence of HPV types 30, 74, 86 and 90 in study population

**Figure 3:**
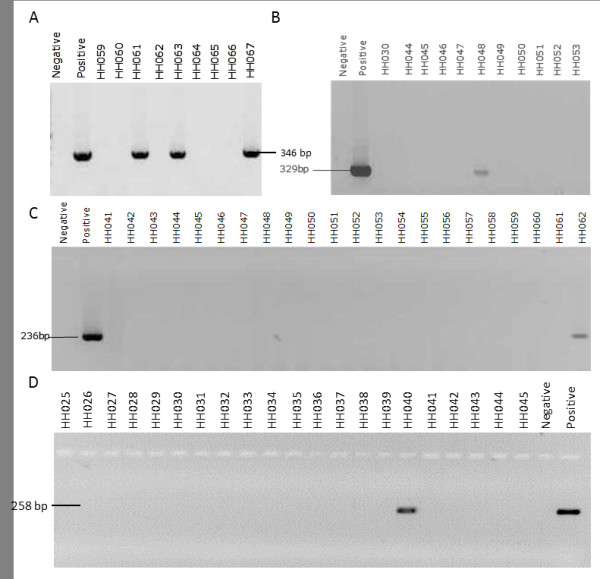
**Representative agarose gel analyses following PCR amplification of a type-specific region within the E6/E7 genes of HPV types 30 (A), 74 (B), 86 (C) and 90 (D) from clinical specimens.** A negative control with water (Negative) and a positive control using DNA from HH015 (Positive) were included.

To confirm the presence of the additional types detected by Illumina sequencing we designed type-specific primers targeting the E6/E7 genes of HPV-30, -74, -86 and −90 (Table 4). BLASTN searches of the nt database, with the primers as query, confirmed their type-specificity. PCR amplification using these primer sets, with DNA extracted from specimen HH015 as template, resulted in amplicons of the expected sizes (Figure 3). The PCR products were sequenced and the presence of all four types confirmed in the HH015DNA. The prevalence of HPV types 30, 74, 86 and 90 in the cohort of 109 HIV positive South African women was then determined by PCR (Figure 3) using the type-specific primers, and found to be 14.6%, 12.8%, 4.6% and 8.3%, respectively.

**Table 4:** Type-specific primers for the detection of HPV types 30, 74, 86 and 90

**HPV type**	**Primer name**	**Sequence**	**Target**	**Amplicon size (bp)**
30	HPV30F	TGAGGTACAAGAAACATCGTTGC	158-180^a^	346
	HPV30R	CGTACGTGATATTCTGTGAAACC	503-481^a^	
74	HPV74F1	TGCTGGACAACATGCATGGAAAAATCCTAC	418-448 ^d^	328
	HPV74R1	CCTCTGTACCTGTATTTTCCGCCATGT	745-719 ^d^	
86	HPV86F	GTTTGTAGGAGTGTGGCATCC	29-10^b^	236
	HPV86R	TTGTACTGCCAAGTTTCTGTCC	7781-7802^b^	
90	HPV90E6F	GCGGAACAGCATTAACAGAGGA	95-116 ^c^	258
	HPV90E6R	TAAGCCGTTCCTTTTCCTCACT	352-331 ^c^	

^a^Position of primers within the E6 gene of HPV 30 (X74474), ^b^HPV 86 (AF349909) and ^c^HPV 90 (AY057438).

^d^Position of primers in the E6 and E7 genes of HPV 74 AE10 subtype (AF436130).

## Discussion

Several studies have indicated that many current HPV typing methods are not able to reliably identify all types present in complex multiple infections [19,21]. In the WHO HPV LabNet Global genotyping proficiency study most labs (90%) were able to identify HPV-16 and 18 as individual types but less than 80% were able to identify HPV 56, 59, and 68. Of more concern was that 28/84 data sets reported false positive results. A notable decrease was observed in the performance of most assays in identifying types when present in multiple infections and only 50 to 73% of the data sets generated by these assays correctly detected the types present [19]. This suggests the need for further assessment of the tests used and regular participation in proficiency testing to ensure the quality of data. It also suggests that epidemiological data may not be completely accurate and result in detection biases.

Recently a study was published using 454 NGS technology and HPV specific primers targeting the conserved L1 gene. A good correlation was reported between INNO-LiPA HPV Genotyping Extra assay (Innogenetics, Gent, Belgium) and NGS data but the NGS had a lower sensitivity [25]. This study differs from our NGS study in that our study does not use specific primers, had a pre-amplification enrichment step using RCA as well as using the Illumina GAII system to generate sequence. Of note was that using this methodology there was a greater sensitivity than LA genotyping. This study demonstrates the use of NGS in genome sequencing and genotyping of the HPV types present in a complex multiple infection in an unbiased manner. The study design was a metagenomic-based approach, extracting total DNA from a cervical specimen (HH015), without prior virus purification. Circular DNA present in the sample was enriched using phage phi29 DNA polymerase in a randomly-primed RCA method [26]. This allowed us to amplify whole HPV genomes in the sample in an unbiased sequence-independent manner, unlike other amplification methods such as PCR. This robust technique has successfully been used for the amplification of a number of circular DNA viruses (reviewed in [27]), including HPV [28], and provides ample quantities of sufficiently pure DNA for sequencing. Illumina sequencing was chosen based on the expected high depth of coverage achieved with this technology.

Approximately 20% of the short sequence reads generated by Illumina sequencing of the RCA-enriched DNA from specimen HH015 were identified as HPV sequences (Figure 2). Considering the small size of the HPV genome (8Kb), even in relatively high copy numbers, in relation to the human genome (ca. 3000 Mb), this level of coverage indicates the highly successful amplification or enrichment of the HPV DNA by the RCA technique. Complete or near complete genomes were assembled for five HPV types (30, 39, 40, 16, 56). Both *de novo* assembly and reference mapping identified these types as being the most abundant in the sample. We were not able to *de novo* assemble full genomes for the less abundant types in the sample, and instead used reference mapping to identify all the types present.

As the HH015 sample contains a mixture of HPV types, reference mapping may be problematic. Short reads sequenced from one type may map to regions of high identity in the genomes of other types present. This could lead to an under or over-estimation of a types’ abundance, or worse, a false positive. This would be particularly dependent on the presence of other highly related types. This is well illustrated when comparing the read count and coverage obtained when we performed read mappings to HPV genomes individually and simultaneously (Table 2). Visual inspection of the read coverage for different regions within the genomes showed unequal read counts. This has, however, been observed in many genome sequencing projects where read coverage is known to be influenced by a number of factors. To overcome these problems, we performed stringent mappings to a highly variable sub-genomic region, the LCR. A consistent read count was obtained for each type whether the mapping was performed individually with a particular HPV type or simultaneously against all types (Table 3). This then allowed for a greater degree of confidence in identifying HPV types that were less abundant. Further support for this, was our finding that the percentage of the genomes or LCRs sequenced did not differ significantly when reads were mapped individually or simultaneously. The relative coverage obtained for the HPV types should reflect the relative viral loads of each type in the specimen. This is assuming that RCA of different types was equally efficient and did not have any amplification biases. Based on the mapping of sequence to the LCR region the type with the highest copy number was HPV-39 followed by HPV types 16, 40, 56, 74, 30, 71, 70, 35, 45, 59, 90, 55, 86, 81, and then finally HPV type 53 as having the lowest copy number.

Roche LA testing of DNA extracted from HH015 identified 12 HPV types (16, 39, 40, 45, 52, 53, 55, 59, 70, 71, 81 and 84). Illumina sequencing could reliably detect 16 HPV types (39, 16, 40, 56, 74, 30, 71, 70, 35, 45, 59, 90, 55, 86, 81, 53), based on *de novo* assembly and reference mapping to HPV genomes and LCR sequences. Both Illumina and Roche LA therefore detected HPV types 39, 16, 40, 45, 53, 55, 59, 70, 71, 74, 81. LA detected HPV-84 and −52 which were not detected by Illumina sequencing. Illumina sequencing identified an additional 6 types not detected by LA; HPV types 30, 35, 56, 74, 86 and 90. The HR types 35 and 56 are included in the LA, while HPV types 30, 74, 86 and 90, are not.

Illumina sequencing covered 88.6% of the complete HPV-35 genome and approximately 85% of the HPV-35 LCR with 99.6% identity to the reference sequence. No reads mapping to HPV-52 were identified. In the LA the probe for HPV-52 can cross-react with HPV-35, -33 and −58. A separate probe for HPV-35 is included in the LA, but was negative for HH015. This may be due to a low viral load in the specimen; however it may also be that HPV-35 was mistyped as HPV-52 in the LA result. In the WHO HPV genotyping global proficiency study, LA testing was found to frequently give false-positive results for HPV-52. As no reads mapped to the HPV-84 LCR the presence of this type, detected by LA, could not be confirmed by Illumina sequencing. A type-specific PCR using HPV-84 specific primers was unable to detect HPV-84 in specimen HH015 (results not shown). HPV-84 may have been mistyped in the Roche LA result.

The HR HPV-56 was identified by Illumina sequencing as one of the dominant HPV types in HH015. The complete genome was assembled at a coverage of 93.9 (Table 1) and mapped at a coverage of 97.4. This type was not detected by LA: this is probably because the limit of detection for HPV-56 in the LA is very high. Eklund et al. [19] report that this type, and HPV-52, are frequently undetected in many HPV genotyping assays, including LA, and their prevalence is probably underestimated in epidemiological studies. This is especially when compared to HPV-16 and −18 prevalence’s, for which most assays have a significantly lower detection limit.

Illumina sequencing identified several HPV types in HH015 not included in the LA (HPV types 30, 74, 86 and 90). HPV-30 has been classified as possibly carcinogenic [6]. While the remaining types are not classified as HR oncogenic types, we wanted to know the frequency of these types in our study population to determine if they were common. HPV-30 (14.6%) and HPV-74 (12.8%) were found to be the third and fourth most common low risk types in our cohort, after HPV-62 (23.9%) and HPV-70 (15.6%) [13]. The prevalence of HPV-86 and 90 was 4.6 and 8.3%, respectively. Although our study population was small (109), the high prevalence of HPV-30 and −74 may warrant their inclusion in future HPV genotyping studies.

Inclusion of HPV types 30, 74, 86 and 90 into our previously reported HPV prevalence data for this cohort [13], showed that only 9.2% of the women had no HPV (10/109), 18.3% had single HPV infection (20/109) and 72.5% had multiple infection (79/109). The high prevalence of multiple HPV infection in this cohort (72.5%) and small sample size limited our ability to assess the impact of the individual HPV types 30, 74, 86 and 90 on the cervical cytology results.

## Conclusions

In this study we show that the use of RCA and Illumina sequencing bypasses or reduces many of the problems associated with PCR-based HPV detection methods, including the requirement for prior sequence information, false positives due to cross-reactivity between types, false negatives associated with low viral loads, and biased amplification that makes identifying all the types present in a multiple infection difficult [19-21]. Many improvements in NGS technologies have occurred since this project was carried out, including paired-end sequencing, multiplexing and increased sequence output. The study identified four HPV types not detected by commercial assays namely HPV-30, 74, 86 and 90. The prevalence of HPV 30, 74, 86 and 90 in 109 HIV positive South African women were found by type-specific PCR to be 14.6%, 12.8%, 4.6% and 8.3% respectively. As these types were identified in a single specimen from an HIV positive woman it is expected that there are many HPV types not being detected in molecular epidemiology studies using commercial kits. The significance of these types in the context of their association to cervical disease remains to be investigated.

## Methods

### Study population and HPV status

The study participants (n = 109) were recruited from women visiting an anti-retroviral (ARV) treatment clinic in Cape Town, South Africa [13]. University of Cape Town Health Sciences Faculty Research Ethics Committee approved the study (Reference 068/2005). Written informed consent was given to take cervical samples for HPV testing. All the women in the study were eighteen years or older and the median age was 31 years (range 20–60 years). The women who were pregnant, had had a hysterectomy or were menstruating at the time of recruitment were excluded from the study. Pap smears were taken using a cytobrush. An adequate pap smear was obtained for 98/109 women, of which 65/98 (66.3%) were abnormal. Atypical squamous cells of undetermined significance (ASCUS) were identified in 15 (15.3%) of the women, low grade squamous intraepithelial lesions (SILs) in 39 (40%), high-grade SILs in 10 (10.2%) and atypical squamous cells-cannot exclude high-grade SIL in 1 (1.0%) [13]. Samples for HPV typing were taken with a Digene cervical sampler (Gaithersburg, MD) and placed in Digene transport medium. The samples were frozen until extracted for HPV DNA analysis using Roche Linear Array HPV Genotyping test (LA) which can detect 37 HPV types. The number of women positive on this test was 97/109. The most common HPV types were HPV 61 (24%), 66 (18%), 58 (17%), 53 (17%), 70 (16%) 45 (16%) and HPV 18 (16%) [13]. Among these, HPV types 66, 58, 45 and 18 are high-risk, HPV-53 is a probable high-risk and HPV-61 and −70 are low-risk types. One specimen, HH015, had 12 HPV types detected by the LA and this specimen was selected for Illumina sequencing.

### DNA extraction and rolling circle amplification

Frozen cervical scrapings stored in Digene transport medium (Qiagen, Gaithersburg, USA) were thawed and total DNA extraction performed using the MagNA Pure Compact Nucleic Acid Isolation Kit I (Roche Diagnostics). HPV circular DNA was enriched for in the extract from specimen HH015 by a RCA method with random hexamer primers, using the IllustraTM TempliPhi kit (Amersham Biosciences) as recommended by the manufacturer.

### Illumina sequencing and data analysis

The DNA from the RCA was fragmented at 32 psi of pressure for 6 min. The fragments were purified using a QIAquick PCR purification spin column (Qiagen, Hilden, Germany). The DNA was subjected to cluster amplification on 1/8 of a flow cell using the Illumina cluster generation kit v2, according to the manufacturer’s instructions. Single read sequencing was performed using the Illumina sequencing kit v3 on an Illumina Genome Analyzer II (GAII) system at the University of Western Cape (Cape Town, South Africa). Illumina sequencing involves the large scale array of DNA templates on a flow cell surface and parallel sequencing of the templates by synthesis, using fluorescently labeled nucleotides that act as reversible terminators during each round of sequencing (http://www.illumina.com/systems/genome_analyzer_iix/technology.ilmn).

The SSRs generated were evaluated with FASTQC (http://www.bioinformatics.bbsrc.ac.uk/projects/fastqc/) and trimmed for quality using FASTx-Toolkit (http://hannonlab.cshl.edu/fastx_toolkit/index.html). Short sequence reads were then *de novo* assembled using Velvet 0.7.31 [23] and CLC Genomics Workbench4.6.1 (CLC bio, Aarhus, Denmark). Velvet *de novo* assemblies were performed with a hash length of 31, and a variety of parameters were tested (expected coverage of 10, 100 or 1000, and coverage cut-offs of 5, 10, or 100) to optimize assembly. Assembled contigs over 100 bases were subjected to BLASTN and BLASTX searches against the NCBI nt and nr databases, respectively, using default parameters and an E value of 10^-5^. SSRs were also mapped against reference HPV sequences obtained from the BLAST searches using CLC Genomics Workbench 4.6.1 (CLC bio, Aarhus, Denmark. Reference mapping was performed with strict mapping settings (global alignment with a mismatch cost of 2 and limit of 3 or 5).

### HPV type-specific PCR

In order to determine the prevalence of HPV types −30, 74, 86 and 90 in cervical specimens from the 109 women in our study population HPV-type specific PCR was performed. Primers were designed to specifically target the E6/E7 genes from HPV types 30, 74, 86 and 90 (Table 4). PCR was performed in a 25 μl reaction volume containing final concentrations of: 1X GoTaq DNA polymerase buffer (Promega, Madison, USA), 1 mM MgCl_2_, 200 μM of each dNTP, 0.65U GoTaq DNA polymerase enzyme (Promega, Madison, USA), 0.4 μM of each primer and 2 μl DNA template. The MgCl_2_ concentration was adjusted to 2 mM for the amplification of the type-specific region of HPV-74. PCR cycling conditions were: 94°C for 10 minutes, 35 cycles consisting of 94°C for 40 seconds, 55°C for 40 seconds, 72°C for 40 seconds, and a final hold at 72°C for 10 minutes. For the amplification of the region of the E6 gene in HPV-86 and −90 the annealing temperatures were adjusted to 53 and 51.5°C, respectively, while the remaining cycling conditions were kept the same. The amplified products were electrophoreses on 2% agarose gels.

## Abbreviations

ARV: Anti-retroviral; Bp: base pair; BLAST: Basic local alignment search tool; HIV: Human immunodeficiency virus; HPV: Human papillomavirus; LA: Roche Linear Array HPV genotyping assay; LCR: Long control region; nt: nucleotide; NGS: Next generation sequencing; PCR: Polymerase chain reaction; RCA: Rolling circle amplification; SSR: Short sequence read; WHO: World Health Organization.

## Competing interests

The authors have no competing interests to declare.

## Authors’ contributions

EPR and ALW initiated the study and participated in supervising all aspects of the study as well as in editing the manuscript. TLM analyzed the data and drafted the manuscript. ATS and TLM performed the experiments. BC, HJM and MJF participated in the Illumina sequence data analysis. JM arranged for collection of the clinical specimens. IIH co-supervised the work done by ATS. All authors read and approved the manuscript.
